# Case Report: Intrapulmonary Arteriovenous Anastomoses in COVID-19-Related Pulmonary Vascular Changes: A New Player in the Arena?

**DOI:** 10.3389/fmed.2021.639152

**Published:** 2021-02-09

**Authors:** Salah D. Qanadli, Ana Carolina Rocha, David C. Rotzinger

**Affiliations:** Cardiothoracic and Vascular Division, Department of Diagnostic and Interventional Radiology, Lausanne University Hospital and University of Lausanne, Lausanne, Switzerland

**Keywords:** COVID-19, computed tomography, perfusion, pulmonary embolism, arteriovenous anastomoses, respiratory failure

## Abstract

Up to now, COVID-19-related vascular changes were mainly described as thrombo-embolic events. A handful of researchers reported another type of vascular abnormality referred to as “vascular thickening” or “vascular enlargement,” without specifying whether the dilated vessels are arteries or veins nor providing a physiopathological hypothesis. Our observations indicate that the vascular dilatation occurs in the venous compartment, and underlying mechanisms might include increased blood flow due to inflammation and the activation of arteriovenous anastomoses.

## Introduction

Early in the coronavirus disease 2019 (COVID-19) pandemic, a high prevalence of vascular disorders has been reported (1). Such abnormalities were mainly described in the lung and covered a broad spectrum of patterns revealed at histology–including microangiopathy, intussusceptive angiogenesis, and microthrombosis–and at imaging with vessel dilatation, tortuosity, thrombosis, and perfusion abnormalities. Up to now, no convincing theory has helped understand the relationship between virus-induced inflammatory disorders and biological and morphological changes, especially those observed on computed tomography (CT). Furthermore, the refractory hypoxemia observed in COVID-19 patients appears to be driven by more complex processes than alveolar damage with low gas exchange alone because COVID-19 leads to severe respiratory failure despite relatively well-preserved lung gas volume (2). This suggests the contribution of vascular phenomena beyond a simple ventilation–perfusion mismatch.

## Vascular Changes in COVID-19 Pneumonia

Imaging-based morphological vascular abnormalities in the lung described at CT may be categorized into three groups: thromboembolic events (3), vascular dilatation, also known as vascular “thickening” or “engorgement” (4, 5), and perfusional changes (6). Mechanisms leading to vascular remodeling remain unclear, and their prevalence and distribution are a matter of debate. We analyzed CT data from a patient who presented all the three groups of abnormalities simultaneously and thoroughly assessed vascular findings, trying to understand the different groups' relationships and elucidate the underlying mechanisms.

**Figure 1:**
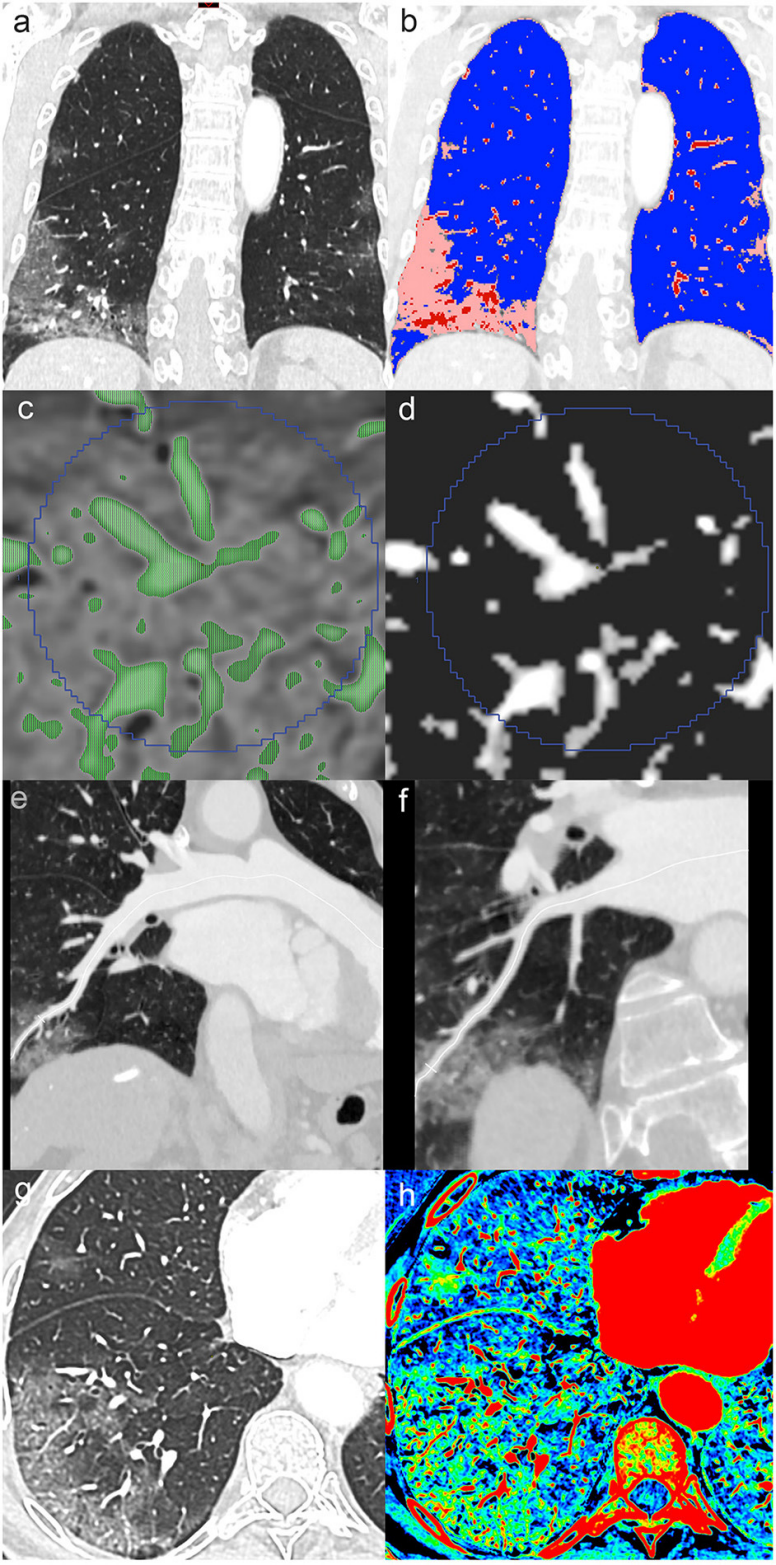
Contrast-enhanced chest CT in a patient admitted for COVID-19. Coronal reformatted image **(a)** shows peripheral ground-glass opacity (GGO) predominantly involving the right lower lobe. Tissue classification **(b)** distinguishing alveolar opacity (peach color code) from normal parenchyma (blue) and vascular components (red) visually indicates vascular enlargement in COVID-19 pneumonia. Specific thresholds to isolate voxels, mostly containing vascular elements **(c,d)**, enable vascular volume extraction in regions-of-interest. Center-line reconstructions of the right posterior basal artery and vein **(e,f)** allow diameter measurement in GGO. Axial conventional image in lung window **(g)** with peripheral COVID-19-related GGO and corresponding dual-energy CT iodine density map **(h)** show increased iodine distribution in GGO consistent with hyperperfusion.

A man in his 70's with fever, tachypnea, bilateral basal crackling sounds, and reverse transcription-PCR (RT-PCR)-proven severe acute respiratory syndrome coronavirus 2 (SARS-CoV-2) infection underwent dual-energy CT pulmonary angiography to rule out pulmonary embolism. Arterial PaO_2_ was 64 mmHg, and SpO_2_ was 92% on room air. The examination was carried out on a fast kV-switching dual-energy CT platform (Revolution CT, GE Healthcare), with the following parameters: rotation speed, 0.5 s; tube load, 180 mAs; reconstructed slice thickness, 1.25 mm; and section interval, 1 mm. Using a power injector, 50 ml of iodinated contrast material (Accupaque 300®) was injected through an 18G venous catheter in the right antecubital fossa at a rate of 4 ml/s and followed by a saline chaser. Findings included zones of COVID-19 ground-glass opacity (GGO) surrounded by healthy parenchyma, enlarged blood vessels within GGO, and acute pulmonary embolism in a lung segment without GGO. We applied automatic tissue classification to distinguish alveolar opacity, normal parenchyma, and vascular components (Figures 1a,b). In a second step, we used a threshold-based automatic segmentation to extract the (macroscopic) intravascular blood volume in a region-of-interest (ROI) in both normal parenchyma and GGO (Figures 1c,d). Calculated intravascular blood volumes showed that in the areas presenting with typical parenchymal changes, the vascular volume was increased by 40% (5.27/300 and 9.0/300 cm^3^ vessel-to-tissue ratio in healthy and GGO zones, respectively). Of note, no venous thrombosis was seen. Furthermore, we demonstrate that the increased volume primarily depended on venous dilatation in the involved lung areas (Figures 1e,f). Arterial and venous diameters at a sub-segmental level in GGO were 3.6 and 4.9 mm, respectively, whereas in the healthy contralateral posterior basal segment, diameters were 3.0 and 3.1 mm, respectively. The corresponding artery-to-vein ratios are 0.97 (3.0/3.1) in healthy parenchyma and 0.73 (3.6/4.9) in GGO, and the vein-to-vein ratio (GGO vs. healthy segment) was 1.58 (4.9/3.1), indicating marked venous enlargement in COVID-19-related GGO. Note that the artery-to-artery ratio (GGO vs. healthy segment) was 1.2 (3.6/3), indicating a moderate arterial dilatation in GGO consistent with hyperemia.

## Discussion

Inflammation-mediated hyperemia is unlikely to be the only factor causing such a marked venous dilatation. We hypothesized that the upregulation of nitric oxide synthase, causing the activation of physiological arteriovenous anastomoses (7, 8) in the involved parenchyma, might explain venous engorgement; these anastomoses create a right-to-left shunt. The existence of pulmonary arteriovenous anastomoses has been suggested and studied by Tobin et al. since the 1950's, and their anatomical location was described as “at the apex of and within the lobular divisions of the lung” (9, 10). Available data suggest that such anastomoses can be activated passively by exercise or supine position, but also actively in the setting of vascular redistribution under both hyperoxia and hypoxia (11). In COVID-19 pneumonia, the consequence of combined mechanisms is exacerbated hypoxia, giving a better explanation for the discrepancies between the relatively preserved ventilation mechanics, the severity of respiratory failure, and the limited response to invasive ventilation (2). Other injuries, such as endotheliitis (12) and/or distal microthrombosis (13), might potentialize the dysregulation of intrapulmonary arteriovenous anastomoses and the resulting shunting effect. In the case we discuss here, transthoracic saline echocardiography would have been a simple and effective means of evaluating the presence of intrapulmonary anastomoses and should have been performed if possible (14). Furthermore, recent evidence suggests that the recruitment of intrapulmonary arteriovenous anastomoses may be driven by the combination of increased cardiac output and increased pulmonary vascular pressure (8). Unfortunately, we could not provide a meaningful estimation of cardiac output based on the available data.

The observed phenomenon is consistent with previously described increased parenchymal perfusion in COVID-19 GGO with dual-energy CT (6). Likewise, our patient exhibited hyperperfusion in GGO zones on iodine density maps (Figures 1g,h). This distal hyperperfusion is attributed to hyperemia induced by the inflammation cascade in COVID-19 pneumonia.

It is also interesting to note that macro-thromboembolic changes (pulmonary embolism) were observed in a different territory than those with parenchymal involvement. This might be another consequence of the vascular shunting effect. This finding is also in agreement with a previous report (15).

In conclusion, our observations indicate that COVID-19-related macroscopic vascular changes depicted *in vivo* are not exclusively due to thromboembolic events. The observed vascular remodeling is mainly related to venous dilatation and might result from combined inflammation-induced hyperemia and dysregulation of arteriovenous anastomoses. Although difficult to establish *in vivo*, vascular shunts could explain the worse than expected clinical course given a relatively modest parenchymal involvement and no visible local thromboembolism. Further studies aiming to characterize those abnormalities in a large series, particularly their distribution and correlation to clinical findings, are needed. The core message of this letter is to frame the hypothesis of intrapulmonary arteriovenous anastomoses as an influencing factor in patients with COVID-19 pneumonia, and the images presented in this case study serve for illustration purposes. An ongoing investigation, the Swiss National Registry COVID-CAVA, is expected to provide relevant insights to address those crucial questions better.

## Data Availability Statement

The original contributions presented in the study are included in the article/supplementary material, further inquiries can be directed to the corresponding author/s.

## Author Contributions

SQ performed the literature research and built the concept. DR prepared the figure. All authors were involved in drafting the manuscript and revising it critically.

## Conflict of Interest

The authors declare that the research was conducted in the absence of any commercial or financial relationships that could be construed as a potential conflict of interest.
